# A Retrospective Comparison of Toxicity, Response and Survival of Intensity-Modulated Radiotherapy Versus Three-Dimensional Conformal Radiation Therapy in the Treatment of Rectal Carcinoma

**DOI:** 10.7759/cureus.48128

**Published:** 2023-11-01

**Authors:** Georgios Kouklidis, Manolis Nikolopoulos, Omer Ahmed, Boulos Eskander, Ben Masters

**Affiliations:** 1 Orthopaedics, University Hospitals Dorset, NHS (National Health Service) UK, Poole, GBR; 2 Gynae-oncology, University Hospitals Dorset, NHS (National Health Service) UK, Poole, GBR; 3 Psychiatry, Dorset Healthcare University, NHS (National Health Service) UK, Poole, GBR; 4 Oncology, University Hospitals Dorset, NHS (National Health Service) UK, Poole, GBR

**Keywords:** pathological response rate, survival, toxicity, three-dimensional conformal radiotherapy, rectal cancer

## Abstract

Introduction: The main target of neoadjuvant treatment in rectal cancer is to downstage and downsize large tumours to increase the chance of complete surgical resection, and therefore decrease the chances of local recurrence. With or without the addition of chemotherapy, until recently, three-dimensional conformal radiotherapy (3D-CRT) used to be the radiotherapy treatment modality of choice. However, intensity-modulated radiotherapy (IMRT) is being increasingly adopted by many radiotherapy centres as a more modern, conformal technique due to its ability to minimize radiation dose to nearby organs. The aim of our analysis was to assess the difference in toxicity, response to treatment, and survival between the patients treated with these two different treatment modalities in our institution.

Methods: We performed a retrospective analysis of data and compared two groups of patients with locally advanced rectal cancer who were treated with either 3D-CRT or IMRT. The main outcomes were radiation toxicity and response to treatment. Overall survival was a secondary outcome.

Results: One hundred and thirty-six patients were included in the study: 71 patients treated with 3D-CRT and 65 patients treated with IMRT. With regard to toxicity, there was no significant difference between the groups for bladder and skin toxicity, but there was a significant reduction in acute grade 2 bowel toxicity in patients treated with a long course of IMRT [3D-CRT 77% (48/62) vs IMRT 64% (30/47) p=0.042]. There was no statistically significant difference in the treatment response rates of these two radiotherapy treatment modalities, as well as in overall survival between the groups (p=0.604). Conclusion: Our study showed that IMRT can significantly reduce acute bowel side effects for patients undergoing neoadjuvant radiotherapy for locally advanced rectal cancers. Further studies are needed to confirm the clinical advantage of IMRT in rectal carcinoma.

## Introduction

The incidence of rectal cancer worldwide was 732,210 with approximately half of these cases resulting in death, in 2020. Amongst all types of cancers, rectal is the eighth most common malignancy with considerable lethality [1].

Despite their histological similarity with other cancers in various locations of the colon, rectal cancers are a disparate entity when it comes to invasion patterns, diagnostic scanning modalities used, as well as surgical treatment options and outcomes [2]. The distinct embryology, anatomy and function of the rectum result in a different treatment approach for primary rectal cancers that require total mesorectal excision (TME) preceded by chemoradiotherapy [3].

The main target of neoadjuvant treatment with either radiotherapy or a combination of chemotherapy and radiotherapy is to downgrade and downsize large tumours and increase the chances of R0 surgical resection, aiming to lower the recurrence rates in the future. Its use is advised for stage T3 or T4 rectal cancer as characterized by pelvic magnetic resonance imaging or endoscopy. Neoadjuvant radiotherapy has been proven to reduce local recurrence rates for locally advanced rectal cancers for many years. Long-course chemo-radiotherapy and short-course radiotherapy treatment courses have been developed. Long-course radiotherapy is generally considered better for downstaging larger tumours; however, it is a five-week course of treatment with the added toxicity of concurrent chemotherapy. Short-course radiotherapy is a much quicker treatment as it is completed in five days but is thought to be associated with increased toxicity. We await the results of the TORPEDO trial to see if this shows a difference between the two techniques [4].

Although it is the standard treatment choice for rectal cancer, side effects and various responses to treatment have been recorded amongst patients. Toxicities, with some of them being solely attributed to radiotherapy, have spurred oncology teams to use new modalities of treatment with better conformality and targeting of the cancer, sparing adjacent healthy tissue. This is how intensity-modulated radiotherapy (IMRT) was introduced. Three-dimensional conformal radiotherapy (3D-CRT) used to be the treatment modality of choice. It uses CT scanning to reconstruct the tumour volume and specifically target it with its beams forming a central cuboid area of high irradiation, which, although precise enough, cannot avoid irradiation to the surrounding healthy tissues. In stark contrast, IMRT, an advanced version of 3D-CRT, with its subdivision of beams into different beamlets with disparate intensity levels creates a very intricate pattern of irradiation that is inhomogeneous. This results in better conformality, bearing a closer resemblance to the target volume shape, as well as titration of doses according to the risk of the irradiated area. Both characteristics entail better protection of healthy tissues [5]. Surveys done in 2016 and 2020 confirmed an increasing trend in the use of IMRT across the NHS, with almost 60% of radiology centres using it as their preferred radiotherapy treatment modality for rectal cancer [6,7]. Various studies assessing dose-related outcomes have concluded that organs in the pelvis, such as the small bowel, bladder and rectum, with the addition of anal sphincters, are better protected from radiation with IMRT rather than 3D-CRT which was the classic treatment modality that was used in the past. Notwithstanding, only a few studies have made a comparison between the clinical outcomes and toxic events of these two treatment modalities. Hence, we took the initiative and organized this retrospective analysis to assess the difference in toxicity, response to treatment, and survival between patients receiving neoadjuvant treatment with IMRT and 3D-CRT in our institution [8,9].

## Materials and methods

We performed a retrospective analysis of data from patients with locally advanced rectal cancer who were treated with neoadjuvant radiotherapy. The inclusion criteria were as follows: (i) pathologically proven rectal adenocarcinoma; (ii) patients receiving preoperative chemoradiotherapy followed by intentional radical resection as well as postoperative, and those that received radiotherapy in the context of palliative treatment; (iii) both locally advanced (clinical T3-4 or nodal involvement) and metastatic diseases at diagnosis; (iv) short- and long-course radiotherapy treatment schedules that were separated out prior to analysis. We compared two groups of patients; one group was treated with 3D-CRT in the years 2018 and 2019 and the other one with IMRT in 2020 and 2021. The main outcomes were the toxicity related to each type of radiotherapy and the response to treatment, assessed with the use of imaging modalities and specifically MRI, CT or PET CT scans. The secondary outcome was the overall survival. To assess the overall survival, we excluded stage IV rectal cancers. This analysis is classified as a service evaluation and is exempt from the requirement for research ethical review according to the current UK research governance arrangements.

Three-dimensional conformal radiotherapy involved a CT planning scan with IV contrast, a radiotherapy plan involving 3-4 fields, and delivering 10 MV photons with daily KV imaging for verification. Long-course radiotherapy involved two phases of treatment, and the phases involved treatment to the pelvis of 45 Gy in 25 fractions followed by an optional 5.4 Gy in three fraction boost to the tumour. Short-course radiotherapy involved a single phase of treatment of 25 Gy in five fractions.

IMRT used a volumetric modulated arc therapy technique, which involved a CT planning scan with IV contrast, and a 6 MV photon treatment plan using 1-2 arcs with daily cone beam imaging for verification. Long-course radiotherapy dose was 45-50.4 Gy in 25-28 fractions and all short-course radiotherapy treatments were 25 Gy in five fractions to the whole volume.

Both 3D conformal and IMRT plans included the rectal tumour, whole mesorectum, internal iliac and pre-sacral lymph nodes. IMRT plans also included the obturator lymph nodes.

Long-course chemotherapy was given with capecitabine 825 mg/m^2^ twice daily 7 days/week throughout the treatment. No chemotherapy was given concurrently with short-course radiotherapy.

Statistical methods

Data were analysed using IBM SPSS Statistics Version 24.0 for Macintosh “macOS” (IBM Corp., Armonk, NY). Results are presented as absolute numbers and percentages for qualitative variables; mean and median values are included for quantitative variables.

Qualitative variables and categorical factors were summarized using frequencies and percentages, whereas quantitative variables and continuous factors were summarized using mean and median values. Fisher’s exact test and chi-square test were used to compare the rate of side effects/toxicities between the two groups of patients. Independent Mann-Whitney U test was used to compare the two groups when more than two variables were present and specifically to compare toxicities categorized according to the organ involved and separated into acute and chronic onset.

Survival curves were generated with the Kaplan-Meier method, and the log-rank test was used to compare survival rates. Overall survival was defined as the time interval between diagnosis and death from any cause. Both cancer-related and non-cancer-related deaths were censored at the time of death for overall survival. p-Values of <0.05 were considered statistically significant.

## Results

We included 136 patients treated with neoadjuvant radiotherapy for rectal cancer. Among these patients, 71 had 3D-CRT within the years 2018-2019 and 65 patients had treatment with IMRT in 2020-2021. The patient demographics, tumour characteristics and treatment types are recorded in Table 1.

**Table 1:** Patient characteristics in IMRT and 3D-CRT groups n: number of patients, %: percentage of patients, 3D-CRT: three-dimensional conformal radiotherapy, IMRT: intensity-modulated radiotherapy, LAR: low anterior resection, APER: abdomino-perineal excision of the rectum, HIPEC: hyperthermic (or heated) intraperitoneal chemotherapy.

Characteristics	3D-CRT, n=71	IMRT, n=65	Total, n=136
Gender	n (%)	n (%)	n (%)
Male	43 (61%)	44 (68%)	87 (64%)
Female	27 (39%)	21 (32%)	48 (36%)
Age	Years	Years	Years
Mean	65.9	70.3	68
Median	65	72	69.5
Range	34-94	34-90	34-94
Grade	n (%)	n (%)	n (%)
1	10 (14%)	1 (1.5%)	11 (8%)
2	43 (61%)	44 (67.5%)	87 (64%)
3	10 (14%)	16 (25%)	26 (19%)
Missing	7 (11%)	4 (6%)	12 (9%)
Stage	n (%)	n (%)	n (%)
1	0	0	0
2	8 (11%)	6 (9%)	14 (10%)
3	40 (56%)	42 (65%)	82 (61%)
4	14 (20%)	12 (18%)	26 (19%)
Type of surgery	n (%)	n (%)	n (%)
Exenterative surgery	36 (51%)	24 (37%)	60 (44%)
LAR	21 (30%)	11 (17%)	32 (24%)
APER	15 (21%)	11 (17%)	26 (19%)
Cytoreductive with HIPEC	0	2 (3%)	2 (1%)
Local excision	2 (3%)	4 (6%)	6 (4%)
Systemic chemotherapy	n (%)	n (%)	n (%)
CAPOX (capecitabine and oxaliplatin)	60 (85%)	45 (69%)	105 (77%)
FOLFIRI (leucovorin, 5-FU, and irinotecan)	0	1 (1.5%)	1 (1%)
FOLFOX (5-FU, leucovorin, and oxaliplatin)	2 (3%)	3 (4.5%)	5 (3.5%)
FOLFOX+Cetuximab	1 (1%)	0	1 (1%)
None	8 (11%)	16 (25%)	24 (17.5%)
Radiotherapy course	n (%)	n (%)	n (%)
Long	62 (87%)	47 (72%)	109 (80%)
Short	9 (13%)	18 (28%)	27 (20%)

There was no statistically significant difference in patient characteristics between the groups comparing age, grade and stage. Most of the patients (109/136, 80%) underwent long-course chemo-radiotherapy rather than short-course one; however, the proportion of patients undergoing short-course radiotherapy was higher, 28% (18/65), in the IMRT group vs 13% (9/71) in the 3D-CRT group, which is likely due to the impact of the COVID-19 pandemic and the pressure to reduce hospital visits during this period. From the 3D-CRT group, 89% (63/71) of the patients underwent chemotherapy but only 75% (49/65) from the IMRT group, which is also likely due to the impact of the COVID-19 pandemic on the use of systemic treatments. In total, 82% (112/136) of patients underwent a course of neoadjuvant or adjuvant systemic chemotherapy prior to radiotherapy or following surgery, respectively, as part of their treatment. The most common regimen used was CAPOX (capecitabine and oxaliplatin) - 77% (105/112).

In the following tables, we recorded our findings of response to treatment for both radiotherapy treatment modalities after separating them into two groups depending on the length of the course used (Tables 2, 3). There were no statistically significant differences found regardless of the treatment modality and the length of course used.

**Table 2:** Response to treatment of patients who had long-course radiotherapy n: number of patients, %: percentage of patients, 3D-CRT: three-dimensional conformal radiotherapy, IMRT: intensity-modulated radiotherapy.

Response to treatment (6-week interval)	3D-CRT, n=62, n (%)	IMRT, n=47, n (%)
Complete	9 (15%)	7 (15%)
Partial	30 (48%)	20 (43%)
Stable/progression	8 (13%)	3 (6%)
Data missing	15 (24%)	17 (36%)
Response to treatment (10-12-week interval)	3D-CRT	IMRT
Complete	9 (15%)	11 (23%)
Partial	22 (35%)	18 (38%)
Stable/progression	6 (10%)	4 (9%)
Data missing	25 (40%)	14 (30%)

**Table 3:** Response to treatment of patients who had short-course radiotherapy n: number of patients, %: percentage of patients, 3D-CRT: three-dimensional conformal radiotherapy, IMRT: intensity-modulated radiotherapy.

Response to treatment (6-week interval)	3D-CRT, n=9, n (%)	IMRT, n=18, n (%)
Complete	0	1 (6%)
Partial	5 (55%)	8 (44%)
Stable/progression	0	3 (17%)
Data missing	4 (45%)	6 (33%)
Response to treatment (10-12-week interval)	3D-CRT	IMRT
Complete	0	3 (17%)
Partial	4 (45%)	7 (39%)
Stable/progression	1 (10%)	0
Data missing	4 (45%)	8 (44%)

**Figure 1:**
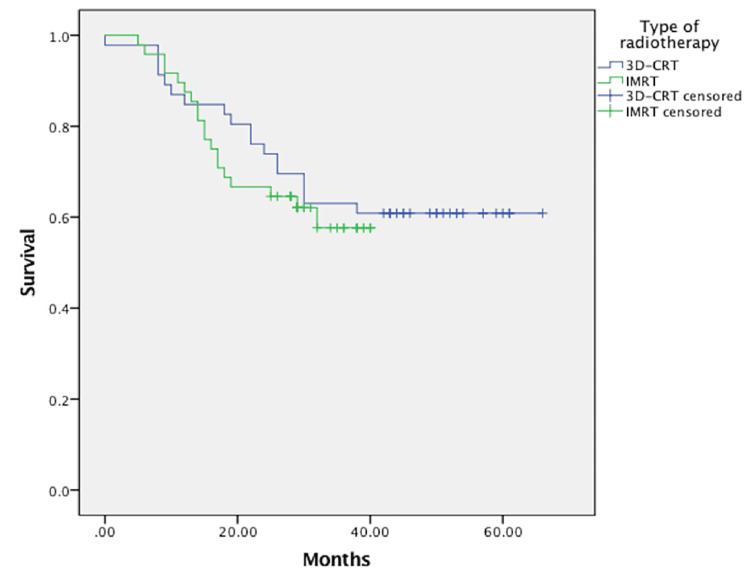
Overall survival plot Not statistically significant p=0.604. 3D-CRT: three-dimensional conformal radiotherapy, IMRT: intensity-modulated radiotherapy.

As seen in the Kaplan-Meier graph, there was no significant difference in the overall survival (p=0.604) between the two groups and there was no change in the timing of deaths either. In the 3D-CRT group, 33 out of 70 patients died and 30 out of 64 in the IMRT group. Two patients have been lost to follow-up (Figure 1).

To compare toxicity, we separated the groups of patients depending on the course of radiotherapy they had. There were very low rates of long-term bladder, bowel or skin toxicities related to the radiotherapy treatment for each radiotherapy treatment modality. There was no significant difference between the two groups when comparing symptoms from bladder, bowel, skin or other organs separately. Patients having acute toxicity in the bowel with long-course radiotherapy showed the biggest difference, favoring treatment with IMRT (59 out of 62 patients treated with 3D-CRT had side effects versus 40 out of 47 treated with IMRT; p=0.056). However, there was a significant difference in grade 2 gastrointestinal (GI) toxicity, when different grades of toxicity were compared separately (p=0.042).

Detailed numbers of acute and chronic toxicity for both long- and short-course radiotherapy are shown in Tables 4, 5.

**Table 4:** Toxicity of long-course radiotherapy n: number of patients, %: percentage of patients, 3D-CRT: three-dimensional conformal radiotherapy, IMRT: intensity-modulated radiotherapy. *Dindo D, et al. [10].

	3D-CRT, n=62	IMRT, n=47
Acute toxicity (Grade*)	n (%)	n (%)
Bladder (p=0.682)		
1	12 (19%)	18 (38%)
2	13 (21%)	8 (17%)
3	0	0
4	0	0
Bowel (p=0.056)		
1	7 (11%)	5 (11%)
2	48 (77%)	30 (64%)
3	4 (6%)	4 (9%)
4	0	1 (2%)
Skin (p=0.481)		
1	3 (5%)	3 (6%)
2	24 (39%)	17 (36%)
3	0	0
4	1 (2%)	0
Chronic toxicity (Grade*)	3D-CRT	IMRT
Bladder		
1	0	0
2	1 (2%)	0
3	0	0
4	0	0
Bowel		
1	0	0
2	0	0
3	3 (5%)	0
4	0	0
Skin		
1	0	0
2	0	0
3	0	0
4	0	0

**Table 5:** Toxicity of short-course radiotherapy n: number of patients, %: percentage of patients, 3D-CRT: three-dimensional conformal radiotherapy, IMRT: intensity-modulated radiotherapy. *Dindo D, et al. [10].

	3D-CRT, n=9	IMRT, n=18
Acute toxicity (Grade*)	n (%)	n (%)
Bladder (p=0.705)		
1	1 (11%)	4 (22%)
2	2 (22%)	1 (6%)
3	0	
4	0	
Bowel (p=0.463)		
1	1 (11%)	6 (33%)
2	4 (44%)	6 (33%)
3	1 (11%)	0
4	0	0
Skin (p=0.495)		
1	0	2 (11%)
2	0	1 (6%)
3	0	0
4	0	0
Chronic toxicity (Grade*)	3D-CRT	IMRT
Bladder		
1	0	0
2	0	0
3	0	0
4	0	0
Bowel		
1	0	0
2	0	0
3	0	0
4	0	0
Skin		
1	0	0
2	0	0
3	0	0
4	0	0

## Discussion

In the last decade, the use of IMRT for the treatment of rectal cancer has skyrocketed from less than half to almost three-quarters of the UK-based radiological cancer centres; the trend is similar in the USA [6,7,11]. Despite this fact, the ASTRO Consensus American Society for Radiation Oncology guidelines (ASTRO) remain controversial with regard to its role, both as neoadjuvant and as adjuvant treatment of rectal cancer [12]. Our study was an attempt to compare the two different radiotherapy treatment modalities for toxicity, complete pathological response and survival and further strengthen the current body of literature regarding this matter.

Our findings of acute grade 2 GI toxicity of long-course radiotherapy, which were documented in 77% of patients treated with 3D-CRT and in 64% of patients treated with IMRT radiotherapy (p=0.042), are in line with Parekh et al. who also found significantly higher toxicity in grade 2 bowel symptoms with 3D-CRT (61% 3D-CRT vs 30% IMRT; p=0.036) [13]. Data from our retrospective analysis are also in agreement with Samuelian et al. showing no significant difference between all other acute or chronic toxicities besides grade 2 acute GI toxicity [67% of 3D-CRT patients experienced grade 2 or above GI toxicity vs 33% of IMRT patients (p = 0.004)] [14]. The Huang et al. study showed slightly different results to our study for acute toxicities higher than grade 2, with significantly reduced acute grade 3 and grade 4 overall toxicity of 8.9% and 20.2% for IMRT and 3D-CRT, respectively [8]. This statistically significant reduction in the IMRT arm was mainly a reflection of the significantly less frequent grade 3 and 4 bowel toxicity. Recorded side effects were diarrhea and proctitis. Our analysis showed that there was no significant difference between grade 3 and 4 acute toxicity for the two arms (6% for 3D-CRT and 11% for IMRT). The difference between the results might be secondary to the exclusion of patients with metastatic disease, new recurrent disease, and history of malignancies other than rectal cancer and the inclusion of only those with pre-operative chemoradiotherapy with the intention to treat. Regarding other toxicities in the acute or chronic presentation, no statistically significant findings were noted, as in our study. A more recent study conducted by Pattanayak et al. published in 2022 also comparing the two different radiotherapy treatment modalities showed similar findings, with the only statistically significant difference being the grade 2 acute toxicity for bowel, which was again less frequent for IMRT [15]. Although a reduction in grade 2 toxicity is not as important as reducing grade 3 or 4 toxicities, it can still have a positive impact on patients and acute oncology services. As many patients with grade 2 bowel toxicity feel unable to venture outside or return to work, it can significantly impact their quality of life. It can also put added pressure on acute oncology services as these patients often contact or attend the acute oncology unit for review and treatment of their symptoms.

Our study did not identify any significant difference in the overall survival between the two modalities of radiotherapy treatment. Our follow-up started in 2018 and continued until 2023 for 3D-CRT, whereas the follow-up of the second cohort of patients that had IMRT started in 2020 and stopped in 2023. Similar findings were also described by Sun et al. among a noticeably bigger sample of 7386 patients and a longer period of eight years as follow-up [16]. Their study only included patients with stage II and III rectal cancer, no other cancer diagnosis in the past, and radiotherapy only as part of neoadjuvant treatment. Our study is also in accordance with their results of response to treatment that showed no statistically significant difference between the two groups of patients: specifically, downstaging of 57% and 55% with 3D-CRT and IMRT, respectively. Jabbour et al. also mentioned no significant difference between the pathologic complete response in their study in 2012 with 20% and 21% as the percentage of complete response for IMRT and 3D-CRT, respectively [17]. In a similar manner, rates of distant metastases and local recurrence bore no difference in the groups of patients receiving 3D-CRT and IMRT. Limitations of their study were the relatively small sample of patients, a short follow-up period of around two years after radiotherapy, and the slight heterogeneity of the chemotherapy received by the patients, concurrently with radiotherapy.

A meta-analysis from 2018 by Wee et al. reached the same conclusion as our study with regard to toxicity and complete response for the two modalities [18]. More specifically, grade 2 or above GI toxicity was observed in significantly fewer patients for IMRT in comparison to 3D-CRT radiotherapy; symptoms that were also assessed separately and following this trend were diarrhea and proctitis. Unlike our study, it also found a statistically significant difference in grade 2 genitourinary toxicity between 3D-CRT and IMRT, favouring the latter. One of the few studies of a prospective nature is a phase II randomized controlled trial by Geary et al., which focused on the comparison of the quality of life of patients with locally advanced rectal cancer undergoing chemoradiotherapy with either IMRT or 3D-CRT [19]. Surprisingly enough, there were only a few quality-of-life benefits of IMRT in comparison to the 3D-CRT arm in this six-year period up to 2020 when the study was terminated. The reason for termination was a failure to establish efficacy in the primary outcome, namely in acute GI toxicity.

Strengths and weaknesses

The fact that we presented the overall survival of patients treated with either 3D-CRT or IMRT is one of the strengths of our analysis, as the evidence available in the literature is limited. Furthermore, compared to other similar studies, our sample size and the length of follow-up time can be considered more than adequate in order to reach some conclusions regarding toxicity, response to treatment and overall survival. The same oncological team treated our patients, namely two different consultants, allowing for only minor differences between the technique and radiotherapy planning.

However, there are some weaknesses in our study, mainly due to its retrospective nature. Risk factors and outcomes could be affected by the fact that the cohort population is limited to the area covered by our NHS trust and the advanced age of the population (mean age 68 years). We included all grades and stages of cancer including those with no intention to treat, such as patients in the palliative setting or with metastatic disease at diagnosis. Additionally, patients who had radiotherapy in both neoadjuvant and adjuvant treatment were included in our sample. It was also not possible to differentiate between toxicity from chemotherapy versus toxicity from radiotherapy in patients who had concurrent chemoradiotherapy from documentation in the notes. Finally, we only evaluated overall survival and not cancer-specific survival to avoid inaccuracies.

## Conclusions

Patients who were treated with IMRT had significantly lesser grade 2 bowel complications and there was a trend towards an improved tumour response rate at 12 weeks; however, this was not deemed statistically significant. Bladder and skin toxicities were found to have no significant difference between the two arms and that was the case at both the acute and the chronic setting. Overall survival analysis also did not show any significant difference between the two arms. Prospective, randomized studies comparing these two modalities of radiotherapy treatment among a sample of patients that allow for safe conclusions to be drawn are required in the future to validate our study.
